# Cardiac Arrest after Local Anaesthetic Toxicity in a Paediatric Patient

**DOI:** 10.1155/2016/7826280

**Published:** 2016-10-31

**Authors:** Liana Maria Torres de Araújo Azi, Diego Grimaldi Figueroa, Ana Amélia Souza Simas

**Affiliations:** Edgard Santos Professor University Hospital, Federal University of Bahia, Salvador, BA, Brazil

## Abstract

We report a case of a paediatric patient undergoing urological procedure in which a possible inadvertent intravascular or intraosseous injection of bupivacaine with adrenaline in usual doses caused subsequent cardiac arrest, completely reversed after administration of 20% intravenous lipid emulsion. Early diagnosis of local anaesthetics toxicity and adequate cardiovascular resuscitation manoeuvres contribute to the favourable outcome.

## 1. Introduction

The caudal block is a procedure often used as a complementary method of analgesia in paediatric surgery. Possible complications of the procedure include intravascular, subarachnoid, or intraosseous injections, all with catastrophic consequences for patients. Intravascular injection occurs in about 1.3 to 10.000 caudal blocks [1].

Overdose toxicity of local anaesthetics has long been known, but its therapy has gained a new ally with the use of lipid emulsions in their treatment [2].

Acceptable theories by which lipid therapy reverses local anaesthetic cardiotoxicity are the “lipid sink” (increasing the clearance of the local anaesthetic from cardiac tissue) and the inhibition of myocardial fatty acid oxidation by the phospholipid [2].

Consent was obtained from the patient for publication of this case.

## 2. Case Presentation

A 6-year-old male child, 22 kg, with congenital glaucoma (using Timolol drops for three years) was posted for hypospadias repair. Uneventful general anaesthesia (GA) induction with fentanyl 100 mcg, Propofol 60 mg, and Rocuronium 15 mg occurred and he was intubated with a 5.5 cuffed. Volume-controlled mechanical ventilation (TV = 200 mL and RR = 18 rpm, PEEP = 5 cmH_2_O, O_2_ 60% in 1,6% sevoflurane) was started and a caudal block in lateral position was performed without difficulty with 12 mL of 0.5% bupivacaine with epinephrine (lack of resistance technique) after negative aspiration for blood or CSF. After supine position, the patient immediately presented hypotension (BP = 60 × 32 mmHg) and cardiac rhythm of ventricular tachycardia with pulse (HR = 176 bpm). Amiodarone hydrochloride 50 mg and epinephrine 20 mcg reverted the rhythm to sinus (HR = 148 bpm, BP = 80 × 45 mmHg). FiO_2_ was raised to 100%. Local anaesthetic intoxication was hypothesized and lipid emulsion was requested. About two minutes later, the patient had cardiac arrest (pulseless ventricular tachycardia interspersed with ventricular fibrillation) and cardiopulmonary resuscitation was initiated. Amiodarone 50 mg + epinephrine 40 mcg was given and a three-time one-minute intravenous bolus injection of 20% lipid emulsion 1.0 mL·kg^−1^ was given, with prompt reversion to sinus rhythm. Then, an intravenous infusion of 20% lipid emulsion at 0.25 mL·kg^−1^·min^−1^ was administered in the next 30 minutes. Surgery was cancelled.

**Figure 1:**
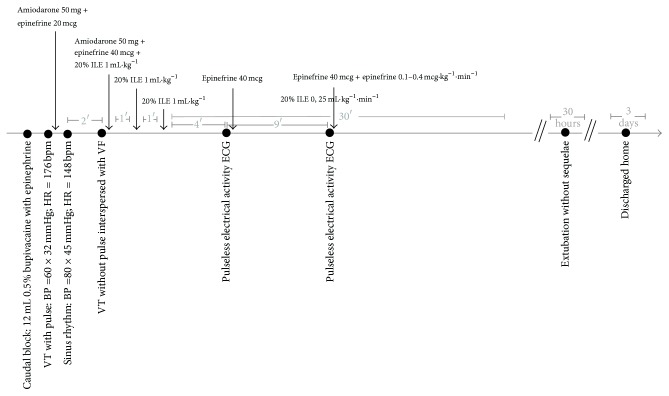


The patient also had two new cardiac arrests in pulseless electrical activity about four and nine minutes later from the previous reversal, treated with effective cardiac resuscitation and 40 mcg bolus of adrenaline. Adrenaline infusion at doses 0.1–0.4 mcg·kg^−1^·min^−1^ was begun. Thereafter, the patient had no further episodes of arrests. Left femoral artery was punctured and urinary catheterization for diuresis monitoring was done. Blood gas analysis showed pH = 7.09, pO_2_ = 86 mmHg, pCO_2_ = 46 mmHg, HCO_3_ = 14 mEq/L, BE = −15.8, and Sat = 93%. Despite respiratory acidosis, lactate concentration was nearly normal (1.7 mmol·L^−1^). He was transferred to the Intensive Care Unit and the adrenaline was turned off after 5 hours of its onset (Figure 1). As perfusion improved, respiratory acidosis disappeared blood lactate level decreased to 0.8 mmol·L^−1^. The patient was kept sedated until normalization of pulmonary and tissue perfusion parameters. Computed tomography of the brain showed no brain damage. He was extubated without sequelae thirty hours after the episode and was discharged home three days later. Formal consent for publication was obtained from patient's mother.

## 3. Discussion

Local anaesthetic toxicity has been reported with almost all kind of regional blocks, including peripheral blockades [3] and caudal blocks [4]. The caudal block is the most easily learned of all regional anaesthetic techniques. It has relatively low risk of complications and a high rate of success. A testing dose can be used to exclude intravascular injection and although desirable it is not commonly performed by most anaesthesiologists. In this case, the ease of injection was used as a parameter for correct placement. The choice of a longer-acting amide LA, such as bupivacaine, improves analgesia after surgery but its cardiotoxicity limits its use [5]. Addition of vasoconstrictors such as epinephrine can dramatically slow its absorption, improving its safety and prolonging the anaesthesia [1]. In this case, the limit dose (3 mg·kg^−1^) was not achieved and the typical description of progressive biphasic symptoms affecting first the CNS and then CVS [6] was not seen, as he was sedated and curarized.

Beta blockade with Timolol drops before surgery may have difficult cardiac arrest reversion and it could also justify the increasing necessities of adrenaline to maintain hemodynamic stable status.

Intravenous lipid emulsion is a method for rescue for cardiovascular collapse after an inadvertent intravenous injection of local anaesthetics [7]. In cardiac arrest caused by bupivacaine intoxication, first-line rescue with epinephrine and epinephrine + intravenous lipid emulsion (ILE) was more effective with regard to survival [8]. There are several case reports of successful resuscitation after cardiovascular collapse in the adult population but examples in children are scarce and this case seems to be the second one showing features of ventilation/perfusion (V/P) mismatch after ILE in child [4]. The doses used in this case followed the ASRA Practice Advisory [9, 10] and resulted in a complete stop of the arrests about 20 minutes after the beginning of its infusion.

If ventilation or perfusion is unstable, a ventilation/perfusion (V/Q) mismatch can occur. It can be caused by blood shunting, for example, during atelectasis, or by dead space in the lungs, for example, with a pulmonary embolism, hypovolemia, or on postarrest period in an intubated and well-ventilated patient. Gas analysis just after cardiac recovery showed respiratory acidosis due to V/Q mismatch. Inadequate pulmonary perfusion (yet) to adequately ventilated areas of the lung impairs gas exchange and leads to hypoxia [11]. FiO_2_ was set to 100% to target SaO_2_ to 90–94% in the postcardiac arrest period.

Despite the fact that it would have resulted in the same consequences (subsequently pulseless electrical activity cardiac arrest), defibrillation using 2 Joules·kg^−1^ would be more suitable than amiodarone as the first choice for treatment of the second cardiac arrest. Induced hypothermia was not performed according to PALS guideline [12].

This case is remarkable because due to the rapid onset of cardiac symptoms intraosseous injection cannot be ruled out. Appropriate conduction of the case favoured a postarrest period without complications.

## References

[1] Giaufré E. (1995). Caudal anesthesia in children. *Cah Anesthesiol*.

[2] Ciechanowicz S., Patil V. (2012). Lipid emulsion for local anesthetic systemic toxicity. *Anesthesiology Research and Practice*.

[3] Kamel I., Trehan G., Barnette R. (2015). Intralipid therapy for inadvertent peripheral nervous system blockade resulting from local anesthetic overdose. *Case Reports in Anesthesiology*.

[4] Shenoy U., Paul J., Antony D. (2014). Lipid resuscitation in pediatric patients—need for caution?. *Paediatric Anaesthesia*.

[5] Lönnqvist P.-A., Morton N. S. (2005). Postoperative analgesia in infants and children. *British Journal of Anaesthesia*.

[6] Mulroy M. F. (2002). Systemic toxicity and cardiotoxicity from local anesthetics: incidence and preventive measures. *Regional Anesthesia and Pain Medicine*.

[7] Ozcan M. S., Weinberg G. (2011). Update on the use of lipid emulsions in local anesthetic systemic toxicity: a focus on differential efficacy and lipid emulsion as part of advanced cardiac life support. *International Anesthesiology Clinics*.

[8] Mauch J., Jurado O. M., Spielmann N., Bettschart-Wolfensberger R., Weiss M. (2012). Resuscitation strategies from bupivacaine-induced cardiac arrest. *Paediatric Anaesthesia*.

[9] Neal J. M., Bernards C. M., Butterworth J. F. (2010). ASRA practice advisory on local anesthetic systemic toxicity. *Regional Anesthesia and Pain Medicine*.

[10] Neal J. M., Mulroy M. F., Weinberg G. L. (2012). American Society of Regional Anesthesia and Pain Medicine checklist for managing local anesthetic systemic toxicity: 2012 version. *Regional Anesthesia and Pain Medicine*.

[11] Donoso F. A., Arriagada S. D., Díaz R. F., Cruces R. P. (2015). Ventilation strategies in the child with severe hypoxemic respiratory failure. *Gaceta Medica de Mexico*.

[12] Kleinman M. E., De Caen A. R., Chameides L. (2010). Special report—pediatric basic and advanced life support: 2010 international consensus on cardiopulmonary resuscitation and emergency cardiovascular care science with treatment recommendations. *Pediatrics*.

